# The risk of SARS‐CoV‐2 infection through sexual contact should be investigated: A timely call

**DOI:** 10.1002/iid3.971

**Published:** 2023-08-08

**Authors:** Syed Masudur Rahman Dewan

**Affiliations:** ^1^ Department of Pharmacy School of Medicine, University of Asia Pacific Dhaka Bangladesh

Dear Editor,

1

In 2019, severe acute respiratory syndrome coronavirus 2 (SARS‐CoV‐2) was first detected in Wuhan, China.^1^ It is one of the most serious health threats currently facing the world. More than 700 million people have contracted this virus to this point, with an estimated 6.5 million succumbing to it so far; the toll is still rising. It has been hypothesized that eating bats exposes humans to coronaviruses, and that respiratory droplets and close contact are the two main routes of transmission for SARS‐CoV‐2.^2^ Even in the absence of symptoms, viruses can be spread through the air by asymptomatic carriers, whose infectiousness is determined by the amount of virus in their upper respiratory tract. Symptoms typically appear after an incubation period of 1−14 days.^3^

In addition, evidence of SARS‐CoV‐2 has been found in the stool, urine, and tear.^4^ The spike protein is the vehicle through which the virus enters the cell. It employs angiotensin‐converting enzyme 2 (ACE‐2) receptors on the surface of targeted cell membranes as cellular receptors to enter the cells, which is followed by the beginning of the life cycle of the virus,^1^ despite the fact that CD147 and NRP1 have also been described as target receptors for the SARS‐CoV‐2 cell entrance.^1^ Therefore, it is reasonable to assume that the listed cell membrane binding site expressing cells, tissues, or more generally organs are most sensitive to the SARS‐CoV‐2 infection. For example, the sources of ACE‐2 receptor expression include the lungs, muscle cells, kidney, ovary, testis, prostate, rectum, colon, adipose tissue, and so forth,^5^ whereas CD147 expressing tissues include the colon, lungs, kidney, ovary, and testis.^6^ These tissues have the potential to be sensitive to viral entry and have the potential to generate COVID‐19 regardless of the severity that is induced.

For our species to survive, sexual behavior must remain constant. When done properly and with caution, it may have a positive effect on both physical and mental health. Today, sex may take many forms, including vaginal, anal, and oral intercourse between bisexual, heterosexual, and homosexual partners. The characteristics of the viruses that cause sexually transmitted diseases make them highly contagious from one partner to another. Each day, more than a million people acquire a sexually transmitted infection (STI), with the vast majority of those cases going unnoticed.^7^ Without adequate cleaning and hygiene, anyone, especially homosexuals and LGBTs (lesbians, gays, bisexuals, and transgenders), can transmit the virus through sharing sex devices. Those who work in the sex industry may also be at high risk for contracting sex‐induced COVID‐19.

The transfer of viruses may occur by saliva, anal‐mucus, vaginal mucus, or viruses on the skin, bear, ears, etc., throughout all stages of sexual contact, not only by vaginal intercourse,^8^ in which the genitalia of both partners contact inside the vaginal canal, is by far the most common and widespread kind of sexual activity. Furthermore, viral traces were identified in the bowel contents^9^ of some patients long after they had healed and there was no evidence of virus in their lungs, which is frightening and highly simple to transfer from one partner to another during oral‐anal sexual stimulation. Assuming the virus is not present in the semen, discovering it in the urine is a means of sexual transmission since the distal urinary and reproductive tracts in males are anatomically connected.^10^

Because both types of receptor proteins are expressed in the testis, prostate, ovaries, and rectum, and because all these organs continue to play a role in human sexual activity, it is impossible to say for certain that infection will not occur via one of these channels. Due to engaging in sexual activity may provide mental calm to both men and women, and because it offers the possibility of stress release, scientists need to give serious consideration to methods of preventing the spread of the disease via sexual encounters. Condom use, global vaccination campaigns, education about STIs, avoiding face‐to‐face contact during sex by trying new sexual positions, good hygiene during sex, avoiding perverted sexual behavior, keeping a safe distance while masturbating, and thorough cleaning and disinfection of sexual implements before and after use have all been cited as effective preventative measures.^11^ However, sex is one of the most exciting parts in the human life which involves both the body and the mind; and maintaining the mentioned criteria before or during sex cannot be possible always, even it may cause unsuccessful sex and may create sexual distance between the partners. Besides, there is no concrete evidence found that only the aforementioned actions could stop the virus from spreading to the partners.

Since the outset, those in charge of public health throughout the globe have been sounding the alarm about the potential for the virus to spread by breathing, touching an item or anything else, and having close contact with one another; additionally, to prevent the spread of the virus, in general, from one person to another, it was necessary to stay away from large groups of people, wash one's hands thoroughly with soap, adequately disinfect one's hands, and wear protective gear. However, we should also exercise extreme caution regarding the SARS‐CoV‐2 viral infection that may be sexually transmittable. Presently, COVID‐19 infection are not being recognized as STI, however, based on the possibilities discussed in this overall article, it is suggested that further molecular‐level research should be conducted in tandem with epidemiological analysis to gain a better understanding of the potential for sexual transmission of SARS‐CoV‐2 as molecular epidemiology is considered to explore the solid disease pathophysiology that ease to develop effective public health policy for disease prevention and treatment.^12^ Since this is a pandemic scenario, it is urgently necessary to determine the actual method that is generally acknowledged as being the most effective at preventing the spread of the virus via this activity to maintain the physical and mental health of human people.

## AUTHOR CONTRIBUTIONS

**Syed Masudur Rahman Dewan**: Conceptualization; formal analysis; project administration; supervision; writing—original draft; writing—review and editing.

## CONFLICT OF INTEREST STATEMENT

The author declares no conflict of interest.
